# Structural Basis of Chemokine Sequestration by CrmD, a Poxvirus-Encoded Tumor Necrosis Factor Receptor

**DOI:** 10.1371/journal.ppat.1002162

**Published:** 2011-07-28

**Authors:** Xiaoguang Xue, Qingyu Lu, Hui Wei, Dongli Wang, Dongwei Chen, Guangjun He, Li Huang, Hanzhong Wang, Xinquan Wang

**Affiliations:** 1 Center for Structural Biology, School of Life Sciences, Ministry of Education Key Laboratory of Protein Sciences, Tsinghua University, Beijing, People's Republic of China; 2 School of Medicine, Tsinghua University, Beijing, People's Republic of China; 3 State Key Laboratory of Virology, Wuhan Institute of Virology, Chinese Academy of Sciences, Wuhan, People's Republic of China; Saint Louis University, United States of America

## Abstract

Pathogens have evolved sophisticated mechanisms to evade detection and destruction by the host immune system. Large DNA viruses encode homologues of chemokines and their receptors, as well as chemokine-binding proteins (CKBPs) to modulate the chemokine network in host response. The SECRET domain (smallpox virus-encoded chemokine receptor) represents a new family of viral CKBPs that binds a subset of chemokines from different classes to inhibit their activities, either independently or fused with viral tumor necrosis factor receptors (vTNFRs). Here we present the crystal structures of the SECRET domain of vTNFR CrmD encoded by ectromelia virus and its complex with chemokine CX3CL1. The SECRET domain adopts a β-sandwich fold and utilizes its β-sheet I surface to interact with CX3CL1, representing a new chemokine-binding manner of viral CKBPs. Structure-based mutagenesis and biochemical analysis identified important basic residues in the 40s loop of CX3CL1 for the interaction. Mutation of corresponding acidic residues in the SECRET domain also affected the binding for other chemokines, indicating that the SECRET domain binds different chemokines in a similar manner. We further showed that heparin inhibited the binding of CX3CL1 by the SECRET domain and the SECRET domain inhibited RAW264.7 cell migration induced by CX3CL1. These results together shed light on the structural basis for the SECRET domain to inhibit chemokine activities by interfering with both chemokine-GAG and chemokine-receptor interactions.

## Author Summary

Chemokines are a family of small proteins that help the immune system fight against invading pathogens by inducing the white blood cells to the areas of infection and inflammation. Due to the important roles of chemokines in immune response, the pathogens evolve diverse mechanisms to neutralize their activities. One example is that large DNA viruses, such as poxviruses and herpesviruses can produce chemokine binding proteins (CKBPs) to sequester chemokines during the infection. The SECRET domain represents a new family of viral CKBPs that was originally identified as a C-terminal extension of the viral tumor necrosis factor receptors (vTNFRs). We determined the three-dimensional structures of the SECRET domain and its complex with chemokine CX3CL1, revealing a new chemokine-binding manner of viral CKBPs. We also showed that other chemokines from different classes may be bound by the SECRET domain in a way similar to that observed in the SECRET/CX3CL1 complex structure. Our biochemical and chemotaxis assays also suggest that the SECRET domain is able to interfere with both chemokine-GAG and chemokine-receptor interactions, both of which are essential for chemokine activities *in vivo*.

## Introduction

Chemokines orchestrate leukocyte migration during immune surveillance, inflammation, and development [1], [2], [3], [4]. They comprise a large family (∼50) of small proteins (∼7–14 KD) that are classified into four classes (C, CC, CXC, and CX3C, where X is any residue) based on the spacing of conserved cysteine residues at the N-terminus [5]. The CC and CXC classes are by far the largest groups of chemokines, whereas the C class consists of two members (XCL1 and XCL2) and the CX3C class contains only one member (CX3CL1). All chemokines share a remarkably similar structural fold, consisting of an extended N-terminus, an antiparallel three-stranded β-sheet and a C-terminal helix [6]. Chemokines exert their biological activities through binding with their cognate G protein-coupled receptors expressed on the surface of leukocytes, as well as binding with endothelial or matrix glycosaminoglycans (GAGs) to form chemokine gradients along which cells travel across endothelium and into tissues [6]. The molecular basis of chemokine-GAG and chemokine-receptor interactions has not been well understood [6], [7]. It has been suggested that the basic residues (typically Arg and Lys) involved in GAG interaction are more or less scattered along the polypeptide chain and form four distinct clusters on the surface of chemokines [8], while the N-termini of all studied chemokines is critical for inducing signaling by their respective receptors [6].

The chemokine network is an important component of host immune response to viral infection [1], [3], which is also extensively modulated by viruses especially large DNA viruses to evade host reactions. Poxviruses and herpesviruses encode their own chemokines, chemokine receptors and chemokine-binding proteins (CKBPs) [9], [10], [11]. The viral CKBPs identified so far are unrelated to any host proteins and exhibit diverse chemokine-binding profiles, reflecting differences in viral tropism and pathogenesis. The viral CC chemokine inhibitor (vCCI, also called T1/35 kDa) secreted by several poxviruses including cowpox virus (CPXV), ectromelia virus (ECTV) and vaccinia virus (VACV) is the most extensively studied, which binds many CC chemokines but not C, CXC, and CX3C chemokines to block chemokine-receptor interaction [12], [13], [14], [15], [16]. The VACV A41 and ECTV E163, representative members of another family of poxviral CKBPs, interact with a subset of CC and CXC chemokines to block chemokine-GAG interaction [17], [18]. Mouse γ-herpesvirus 68 encodes a unique CKBP named as the M3 protein that is able to bind chemokines from the C, CC, CXC, and CX3C classes [19], [20]. Structural and biochemical studies revealed that M3 disrupts both chemokine-receptor and chemokine-GAG interactions [21], [22], [23]. Other viral CKBPs, such as M-T7 from myxoma virus (MYXV), a CKBP from orf virus (ORFV), p21.5 from human cytomegalovirus and glycoprotein G from α-herpesviruses, have also been described previously [24], [25], [26], [27].

Four different genes encoding viral tumor necrosis factor receptors (vTNFRs) have been identified in poxviruses, consisting of cytokine response modifier B (CrmB), CrmC, CrmD, and CrmE [10]. They contribute to pathogenesis of poxviruses and reflect the complex regulation of TNF-mediated host immune response [28]. In addition to the anti-TNF activity attributed to the N-terminal four cysteine-rich domains (CRDs) homologous to host TNF receptors [29], CrmB and CrmD have anti-chemokine activity attributed to a unique C-terminal extension (∼160 aa), named as the SECRET domain (smallpox virus-encoded chemokine receptor) [30]. Biochemical analysis revealed that the SECRET domain binds a subset of human and mouse CC, CXC and C chemokines, including CCL28, CCL25, CCL20, CXCL12, CXCL13, CXCL14, and XCL1 [30]. The identification of other poxvirus genes encoding homologues with the SECRET domain indicates that the SECRET domain represents a new family of viral CKBPs, which has specific folding to allow its binding with chemokines, either independently or fused with vTNFRs [30], [31]. A recent report predicted the structural homology of the SECRET domain with CPXV vCCI and VACV A41 and also analyzed its structural differences from vCCI and A41 based on a *de novo* model [32]. Here we report the crystal structures of the SECRET domain of CrmD encoded by an ECTV strain [33] and the complex of it with chemokine CX3CL1. These structures, together with biochemical and chemotaxis assays, reveal the structural basis for the SECRET domain to bind chemokines and also shed light on its anti-chemokine structural mechanisms.

## Results

### Structure of the SECRET domain

**Figure 1:**
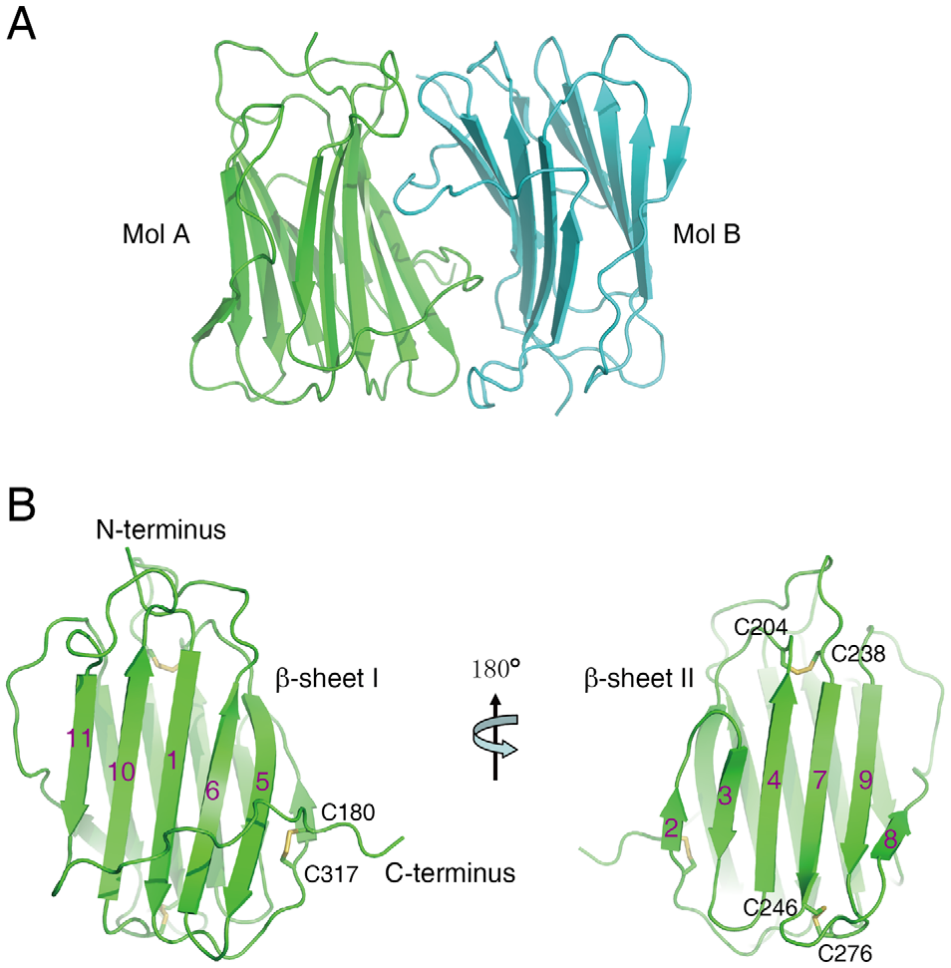
Crystal structure of the SECRET domain. (A) Ribbon diagram of two SECRET domain monomers in the asymmetric unit. (B) Ribbon diagram of the SECRET domain monomer showing the β-sheet I (left) and β-sheet II (right).

The crystal structure of the SECRET domain (residues S162−D320) was determined at a resolution of 1.57 Å by using single-wavelength anomalous dispersion (SAD) method with a Br-soaked derivative (Table 1 and Figure S1 in Text S1). There are two SECRET domains (molecules A and B) in the asymmetric unit (Figure 1A), related by a non-symmetrical two-fold axis with an r.m.s.d. of 0.62 Å for all Cα atoms. Although these two monomers bind each other tightly with a buried surface of ∼1160 Å^2^, the size exclusion chromatography revealed that it is monomeric in solution (Figure S2 in Text S1). The same phenomenon was also observed in the CPXV and ECTV vCCI crystal structures [34], [35]. Therefore, the SECRET dimer in the asymmetric unit is caused by molecular packing and unlikely has any functional significance.

**Table 1:** Crystallographic statistics.

	Native SECRET	Br-soaked SECRET	SECRET/CX3CL1
**Data collection**			
Beamline	SSRF BL-17U	SSRF BL-17U	SSRF BL-17U
Wavelength	0.9793	0.9195	0.9795
Space group	C222_1_	C222_1_	P3_2_21
Cell dimensions			
*a, b, c* (Å)	72.42, 73.44, 112.41	72.42, 73.42, 112.55	71.33, 71.33, 93.14
α, β, γ (°)	90, 90, 90	90, 90, 90	90, 90, 120
Resolution (Å)	50–1.57 (1.61–1.57)	50.0–1.46 (1.49–1.46)	50.0–2.60 (2.69–2.60)
*R*_merge_ (%)	7.8 (39.8)	5.7 (24.9)	10.0 (86.7)
*I*/*σI*	17.9 (2.4)	58 (9.7)	17.4 (1.8)
Completeness (%)	98.3 (87.6)	100 (100)	99.9 (99.3)
Redundancy	3.9 (3.2)	9.5 (9.0)	7.1 (6.4)
**Refinement**			
Resolution (Å)	30.7–1.57		30.0–2.60
No. Reflections	39277		8262
*R*_work_/*R*_free_ (%)	16.4/19.9		19.6/25.0
No. atoms			
Protein	2493		1743
water	466		28
B-factors (Å^2^)			
Protein	20.4		75.6
water	32.7		61.7
R.m.s. deviations			
Bond lengths (Å)	0.006		0.010
Bond angles (°)	1.130		1.282
Ramachandran plot			
Most favored	88.4		81.1
Allowed	10.9		16.5
Generally allowed	0.3		1.9
Disallowed	0.3		0.5

The SECRET domain monomer adopts a β-sandwich fold, consisting of two parallel β-sheets and the connecting loops (Figure 1B and Figure S3 in Text S1). The β-sheet I consists of five anti-parallel strands 1, 5, 6, 10 and 11 (Figure 1B and Figure S3 in Text S1). The β-sheet II consists of six strands, which can be further divided into two segments (antiparallel strands 2, 3, 4 and 7; antiparallel strands 8 and 9) (Figure 1B and Figure S3 in Text S1). The β-sheet II outside surface is completely exposed to solvent (Figure 1B), whereas the solvent accessibility of β-sheet I outside surface is limited by a long C-terminal loop after strand 11 surrounding the bottom half of β-sheet I (Figure 1B). A disulfide bond, C180−C317, further fixes the conformation of this extended loop by connecting it to the 1–2 loop (Figure 1B and Figure S3 in Text S1).

### Structural comparison with other poxviral CKBPs

**Figure 2:**
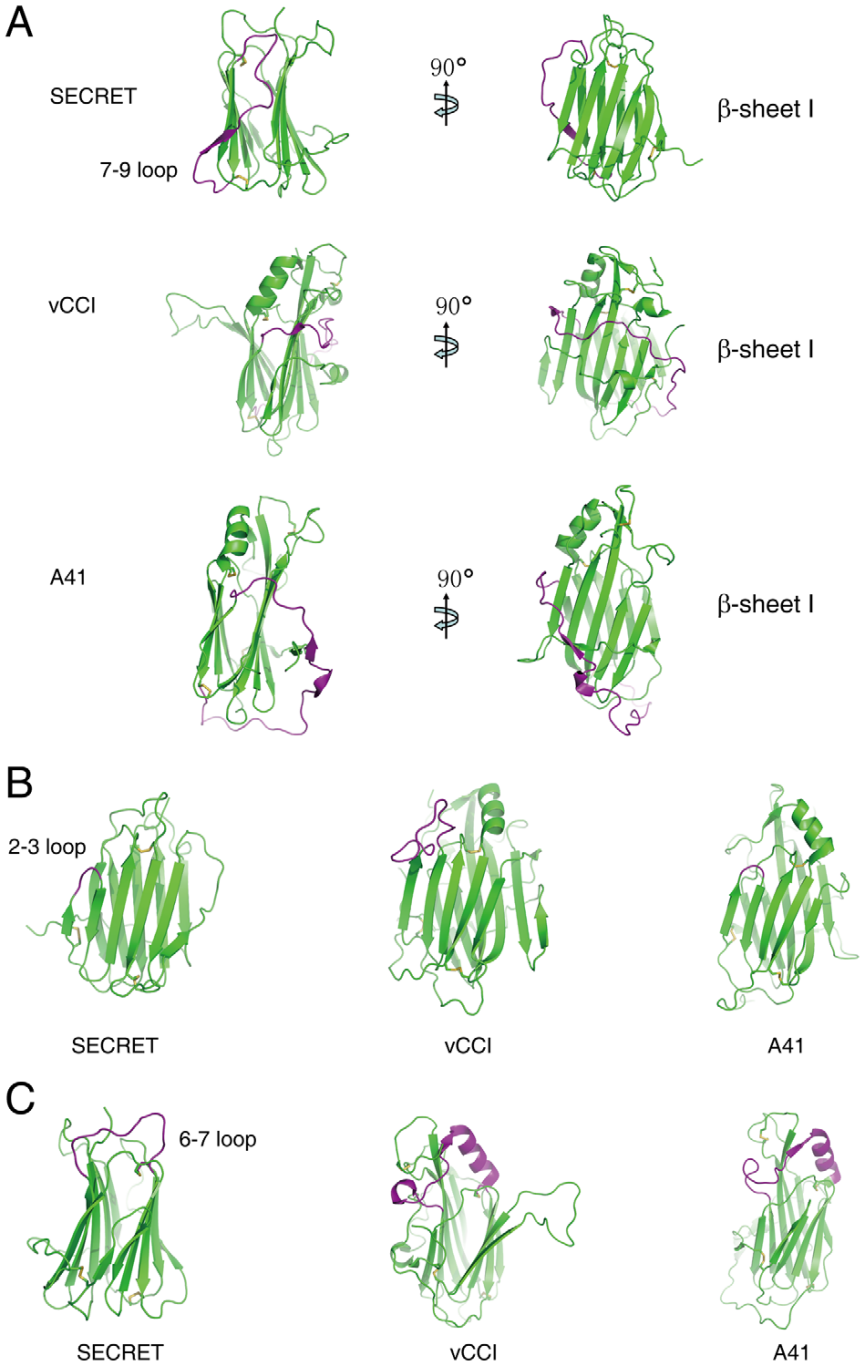
Structural comparison of the SECRET domain with vCCI and A41. (A) The conformational change of the 7–9 loop colored with purple. (B) The conformational change of the 2–3 loop colored with purple. (C) The conformational change of the 6–7 loop colored with purple.

The overall β-sandwich topology of the SECRET domain is similar to that of vCCI and A41 [17], [34], [35], [36]. However, there are several significant differences in the arrangement of certain secondary structure elements, making the CrmD SECRET domain more compact than vCCI and A41 and also directly affecting its binding with chemokines. In the following comparison and description, we use the structure of vCCI from ECTV as the representative member of the vCCI family [35]. The first difference is at the 7–9 loop (S248−H266) in the SECRET domain, corresponding to residues S140 to I168 in vCCI and E113 to M144 in A41 (Figure 2A and Figure S4 in Text S1). The long S140−I168 loop in vCCI wraps the β-sheet I at the top half, and the long E113−M144 loop in A41 wraps the whole β-sandwich from bottom side (Figure 2A). In collaboration with the conserved C-terminal loop surrounding the bottom half of β-sheet I, these two long loops further limit the solvent exposable surface of β-sheet I in vCCI and A41, respectively (Figure 2A). The 7–9 loop in the SECRET domain goes up and down at the β-sheet II side of the β-sandwich, and residues S252 to Q254 form the strand 8 in β-sheet II (Figure 2A and Figure S4 in Text S1). Therefore, it does not limit the solvent exposable surface of β-sheet I in the SECRET domain. The second difference occurs at the 2–3 loop (I184−S186) in the SECRET domain, whose length is nearly the same as that in A41 (K39−Y40) and much shorter than that in vCCI (S52−P66) (Figure 2A and Figure S4 in Text S1). The third difference occurs at the 6–7 loop, whose length in the SECRET domain (N227−C238) is also much shorter than that in vCCI (S107−C131) and A41 (S80−C104) (Figure 2A and Figure S4 in Text S1). There is an α-helix in this loop region of vCCI and A41, which is absent in the SECRET domain (Figure 2A).

**Figure 3:**
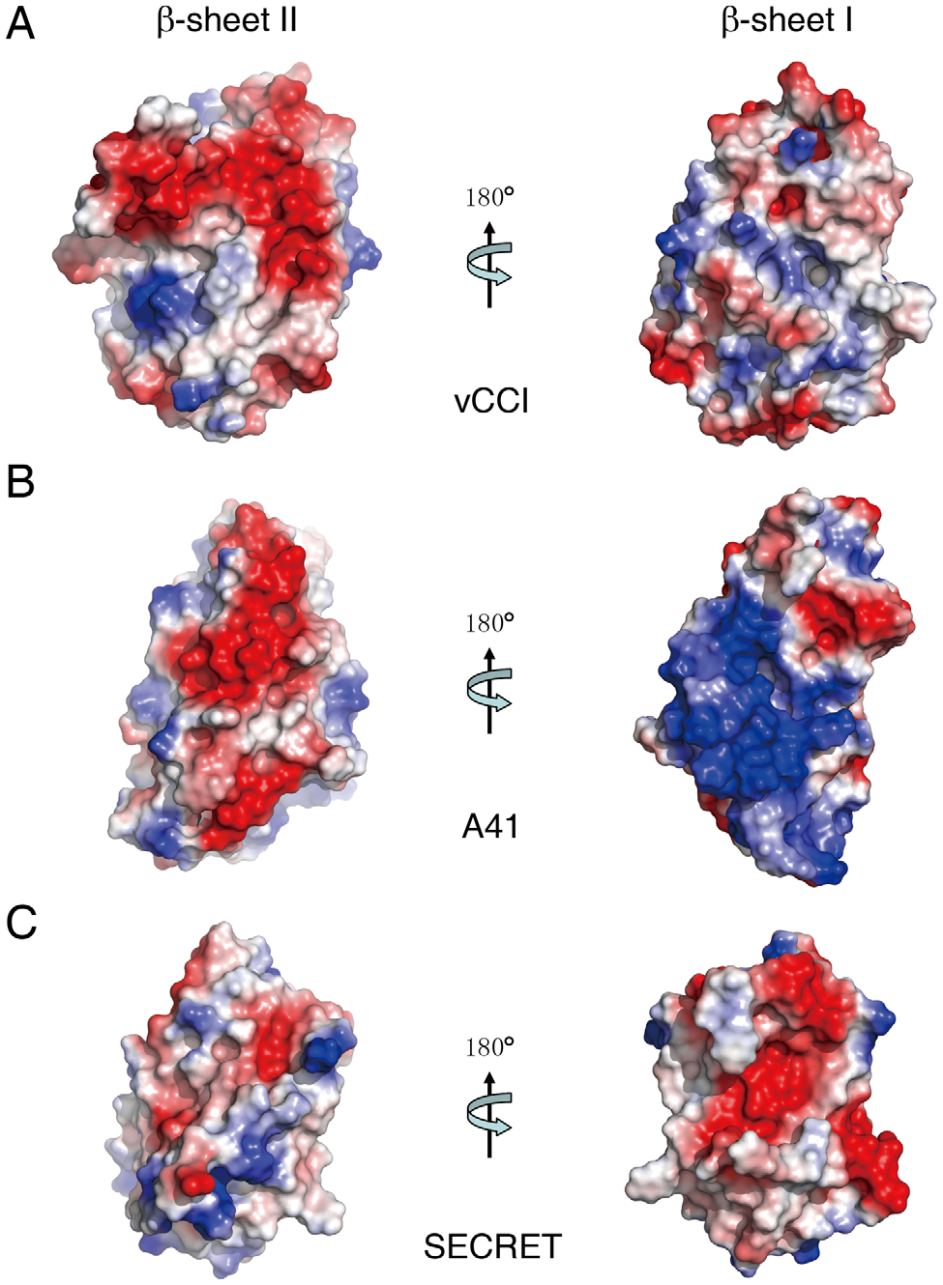
Electrostatic potential surfaces. (A) vCCI. (B) A41. (C) SECRET domain.

The electrostatic complementarity plays a critical role in the binding of chemokines by vCCI and A41 [17], [34], [35], [36]. The β-sheet II of vCCI exhibits strong electronegative character. Negative charge patches, including the protruded acidic 2–3 loop (S52−P66) (Figure 3A and Figure S4 in Text S1), are involved in the interactions with positive charged residues of bound chemokine as revealed in the NMR solution structure of vCCI in complex with chemokine CCL4 [36]. The 2–3 loop (K39−Y40) in A41 is much shorter than that in vCCI, but its β-sheet II also exhibits negative charge patches (Figure 3B and Figure S4 in Text S1) and may contribute to the interaction with bound chemokine [17]. The opposite β-sheet I of vCCI and A41 is comparatively uncharged and electropositive, respectively (Figure 3A and 3B). The SECRET domain exhibits different electrostatic surface by switching the surface charge property as observed in vCCI and A41. Its β-sheet II has no remarkable electrostatic properties, while the opposite β-sheet I exhibits strong negative charge in the solvent exposable region, contributed by acidic residues D167, E169, D228, D290, D316, and E318 (Figure 3C and Figure S4 in Text S1). The distinct surface charge property of the SECRET domain leads us to speculate that it may bind chemokines in a different manner by using the solvent exposable and negatively charged surface of β-sheet I.

### Structure of the SECRET/CX3CL1 complex

To directly elucidate the chemokine binding by the SECRET domain, we reconstituted a complex of the SECRET domain with the chemokine domain of CX3CL1 and determined its structure at a resolution of 2.6 Å. The structure was solved by the molecular replacement method using the SECRET domain and CX3CL1 structures as search models, and refined to final *R_work_* and *R_free_* factors of 19.6% and 25.0%, respectively (Table 1 and Figure S1 in Text S1).

**Figure 4:**
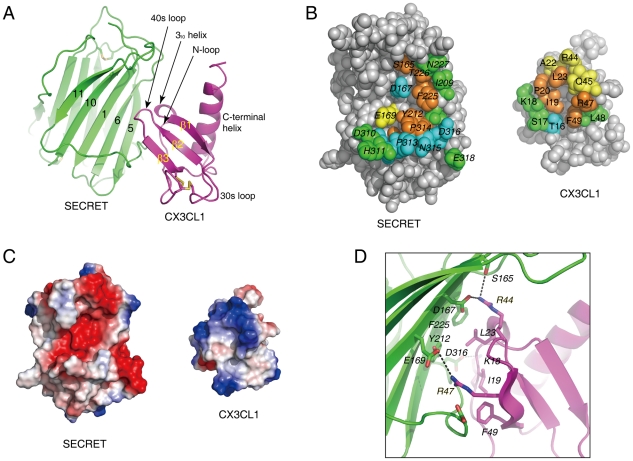
Crystal structure of the SECRET/CX3CL1 complex. (A) Ribbon diagram of the SECRET domain (green) in complex with CX3CL1 (purple). (B) Residues at the binding interface. Residues with solvent accessible surface decrease of above 80%, 60–80%, 40–60%, and below 40% upon complex formation are colored in brown, cyan, yellow, and green, respectively. (C) Electrostatic complementarity at the binding interface. The SECRET domain and CX3CL1 are in the same orientation as in panel B. D) Detailed view of the interactions at the binding interface. Hydrophilic interactions around basic residues K18, R44, and R47 of CX3CL1 are shown as dashed lines.

In the complex, one SECRET domain monomer binds one CX3CL1 monomer, displaying a 1∶1 stoichiometry (Figure 4A). The chemokine domain of CX3CL1 in the complex adopts the typical chemokine-fold topology, consisting of an extended N-loop (C8−P20), a short 3_10_ helix (V21−L23), a 3-stranded anti-parallel β-sheet (β1, L24−Q29; β2: I39−T43; β3: R47−A51), a C-terminal helix (Q56−A69) packing against the β-sheet, and the 30s loop (N30−A38) and 40s loop (R44−H46) connecting the strands in the β-sheet (Figure 4A). The N-terminal residues Q1 to K7 and C-terminal residues R74 to G76 are disordered in the structure. The SECRET domain contacts the CX3CL1 with its β-sheet I, burying a surface of ∼530 Å^2^ (Figure 4A). The SECRET domain contacting residues are from the strands 1, 5, and 6 of β-sheet I and the C-terminal extended loop, while the CX3CL1 contacting residues are from the N-loop, 3_10_ helix, 40s loop, and the β3 strand (Figure 4A). The binding interface can be described as a small hydrophobic core surrounded by a large halo of hydrophilic interactions. The hydrophobic core is composed of residues Y212 and F225 from CrmD, and I19, L23 and F49 from CX3CL1 (Figure 4B). The surrounding hydrophilic interactions are composed of hydrogen bonds and salt-bridges. There is an obvious electrostatic complementarity between the SECRET domain and CX3CL1 at the interface (Figure 4C). The acidic residues D167, E169, and D316 from the SECRET domain form salt-bridge interactions with R44, R47, and K18 from CX3CL1, respectively (Figure 4D).

To further elucidate the roles of important residues in complex formation, we mutated hydrophobic residues I19A, L23A and F49A and charged residues K18A, R44A, and R47A in CX3CL1, and measured the binding affinities of these mutants with the SECRET domain using surface plasmon resonance (SPR) method. We performed two independent measurements for each protein sample and the results are listed in Table 2. The SECRET domain interacted with CX3CL1 with an affinity of 0.68±0.26 µM (Table 2 and Figure S5 in Text S1) The CXC3L1 mutants I19A,L23A, and F49A bound the SECRET domain with affinities of 0.96±0.32, 4.49±1.13, and 4.20±0.45 µM, respectively (Table 2 and Figure S5 in Text S1). The CX3CL1 mutants K18A, R44A, and R47A bound the SECRET domain with affinities of 10.9±0.6 µM, 16.15±0.45 µM, and 36.45±1.95 µM, respectively (Table 2 and Figure S5 in Text S1). All mutations resulted in the decrease of the binding affinity between the SECRET domain and CX3CL1. Mutating charged residues K18, R44, and R47 in CX3CL1 induced more significant binding affinity decrease than mutating hydrophobic residues I19, L23, and F49, suggesting the importance of the salt-bridge interactions in the complex formation of the SECRET domain with CX3CL1.

**Table 2:** Binding measurements of the SECRET domain with CX3CL1 wild type and its mutants by surface plasmon resonance (SPR) analysis.

	Fit model	First measurement			Second measurement			K_D_ (µM)	
		K_D_ (µM)	k_on_ (M^−1^s^−1^)	k_off_ (s^−1^)	K_D_ (µM)	k_on_ (M^−1^s^−1^)	k_off_ (s^−1^)	Mean	SE of mean
WT	Steady state	0.421			0.937			0.68	0.26
K18A	Kinetics	11.5	0.45e3	5.18e-3	10.3	1e3	10.3e-3	10.9	0.6
I19A	Kinetics	1.28	5.38e3	6.89e-3	0.641	9.02e3	5.78e-3	0.96	0.32
L23A	Steady state	3.36			5.62			4.49	1.13
R44A	Steady state	15.7			16.6			16.15	0.45
R47A	Steady state	34.5			38.4			36.45	1.95
F49A	Steady state	3.75			4.64			4.20	0.45

### Comparison with other CKBP/chemokine complexes

The SECRET/CX3CL1 and previous reported vCCI/CCL4 complexes [36] are different in the association manner between CKBP and chemokine, binding interface, and the role of electrostatic complementarity in complex formation. The SECRET domain utilizes its β-sheet I to interact with CX3CL1, whereas vCCI utilizes its β-sheet II to interact with CCL4 upon complex formation. The vCCI/CCL4 binding interface, burying a total surface of ∼990 Å^2^, can be divided into two patches. The patch 1 between the N-loop of CCL4 and vCCI is primarily composed of hydrophobic interactions around CCL4 residue F13 and salt-bridge interactions around CCL4 residue R18 (Figure S6 in Text S1A) [36]. These two positions are conserved in CC chemokines and mutation of them dramatically decreased the binding of CC chemokines by vCCI [37], [38]. The patch 2 is between the basic 40s loop of CCL4 and the extended and acidic 2–3 loop of vCCI (Figure S6 in Text S1A), and the electrostatic complementarity is expected to drive the interactions between them, although basic residues K45, R46, and K48 in the 40 s loop of CCL4 were mutated to Ala in the vCCI/CCL4 complex structure (Figure S6 in Text S1A) [36]. The smaller SECRET/CX3CL1 interface (∼530 Å^2^) is composed one contact patch with a small hydrophobic core and surrounding hydrophilic interactions as described above (Figure 4B). The region from C8 to M15 of the N-loop is far away from the SECRET domain, so the SECRET domain does not utilize critical hydrophobic interactions observed in the vCCI/CCL4 contact patch 1 to bind CX3CL1. The corresponding position of R18 in CCL4 is K18 in CX3CL1, which forms salt-bridge interaction with D316 of the SECRET domain to surround the hydrophobic core (Figure 4D) and is also important for their binding (Table 2). Structural superimposition based on bound chemokines revealed that the smaller SECRET/CX3CL1 interface corresponds to contact patch 2 in the vCCI/CCL4 interface (Figure S6 in Text S1A). Although obvious electrostatic complementarity is observed in both the SECRET/CX3CL1 interface (Figure 4C) and the contact patch 2 in the vCCI/CCL4 interface (Figure S6 in Text S1A), the electrostatic interactions around the basic 40s loop of bound chemokine play a different role in the formation of these two complexes. Residues R44 and R47 of CX3CL1 are critical because mutations at these positions caused respective ∼24-fold and ∼54-fold drop in the binding of CX3CL1 by the SECRET domain (Table 2). In contrast, a triple mutant of CCL4 (K45A/R46A/K48A) had nearly the same binding affinity as wild type CCL4, as determined by ELISA method [36]. In CCL2, the K49A mutation even increased its binding affinity with vCCI [37], [38]. Therefore, the contact patch 2 around the 40s loop of chemokines might contribute to chemokine binding of vCCI by providing an electronegative platform to recruit different CC chemokines, while the conserved hydrophobic and hydrophilic interactions around the N-loop of CC chemokines in the contact patch 1 determine the high affinity binding of CC chemokines by vCCI. The contact patch around the basic 40s loop of CX3CL1 has dual roles, not only helping the recruitment of a subset of chemokines from different classes by the SECRET domain, but also providing critical interactions for the complex formation.

Parasites, such as blood-sucking ticks and *Schistosoma mansoni*, also secrete CKBPs with anti-inflammatory activities [39], [40], [41]. Evasin, a new family of CKBPs encoded by ticks, comprises four members that may help inhibit chemokine-mediated host innate immune responses [40]. In contrast to most of the viral CKBPs, Evasin-1 is very restrictive by only binding CCL3, CCL4 and CCL18 [40]. The Evasin-1 adopts a novel fold and it interacts with bound CCL3 by primarily contacting its N-loop region, as revealed in the Evasin-1/CCL3 complex structure (Figure S6 in Text S1B) [42]. Structural superimposition based on bound chemokines revealed that the SECRET-binding and Evasin-1-binding epitopes on chemokines are distinct with little overlap (Figure S6 in Text S1B). Therefore, the SECRET domain and Evasin-1 are different in the chemokine-binding manner.

The M3 encoded by murine γ-herpesvirus 68 functions as a dimer in solution, in contrast to other monomeric poxviral CKBPs. The two M3 monomers are arranged in a “head-to-tail” manner, each monomer consisting of the N-terminal domain (NTD) and C-terminal domain (CTD) (Figure S6 in Text S1C) [21]. Unlike the SECRET/CX3CL1 complex in 1∶1 stoichiometry, the M3 dimer utilizes the NTD of one monomer and the CTD of the other monomer to form two clefts to bind two chemokines, forming a complex in 2∶2 stoichiometry (Figure S6 in Text S1C) [21], [23]. The SECRET domain and the NTD of M3 have positional overlap around the 40s loop of bound chemokine (Figure S6 in Text S1C). The Evasin-1 and the CTD of M3 have positional overlap around the N-loop of bound chemokine (Figure S6 in Text S1C). Therefore, M3 seems to combine different chemokine-binding manners of the SECRET domain and Evasin-1 by utilizing both NTD and CTD in the binding of chemokines.

### The SECRET domain interferes with the interaction of CX3CL1 with both GAG and cellular receptor

**Figure 5:**
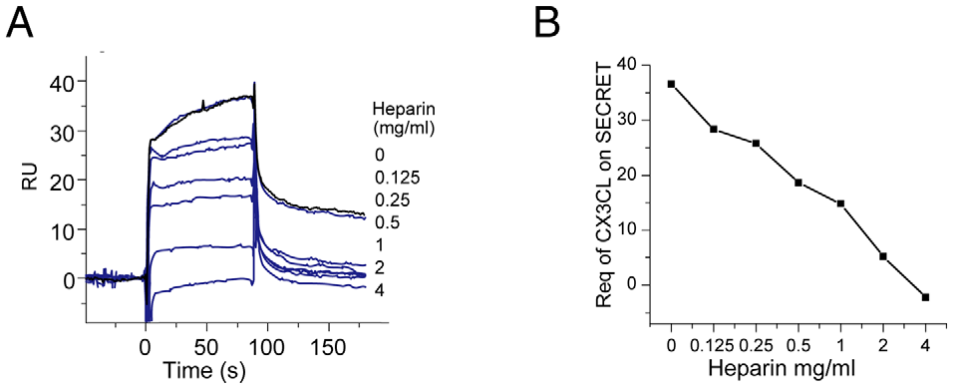
The SPR analysis of SECRET-CX3CL1 interaction in the presence of heparin. (A) Sensorgrams of passing CX3CL1 pre-incubated with an increased concentration of heparin (sodium salt, molecular weight ∼15 KD) (0, 0.125, 0.25, 0.5, 1, 2, and 4 mg/ml) through the CM5 chip surface immobilized with the SECRET domain. The binding of CX3CL1 without heparin pre-incubation (0 mg/ml) by the SECRET domain was checked again (response curve in black) after finishing all other measurements to make sure that the decrease of response was not due to the change of chip surface. (B) Heparin interferes with the binding of CX3CL1 by the SECRET domain in a dose-dependent manner.

We conducted a SPR experiment as reported for other viral CKBPs (VACV A41 and ECTV E163) to test if the SECRET domain can interfere with the interaction of CX3CL1 with GAG [17], [18]. The CX3CL1 was pre-incubated with various amount of heparin (sodium salt, molecular weight ∼15 KDa), and then injected over the SECRET-coupled sensor chip. Heparin decreased the binding of CX3CL1 by the SECRET domain in a dose-dependent manner (Figure 5), indicating the overlap of SECRET-binding and GAG-binding sites on CX3CL1. In our experiment, the concentration of heparin required for the inhibition was much higher than that used to achieve the disruption of chemokine binding by A41 and E163 [17], [18]. This may be caused by the reported low binding affinity between CX3CL1 and heparin [23].

**Figure 6:**
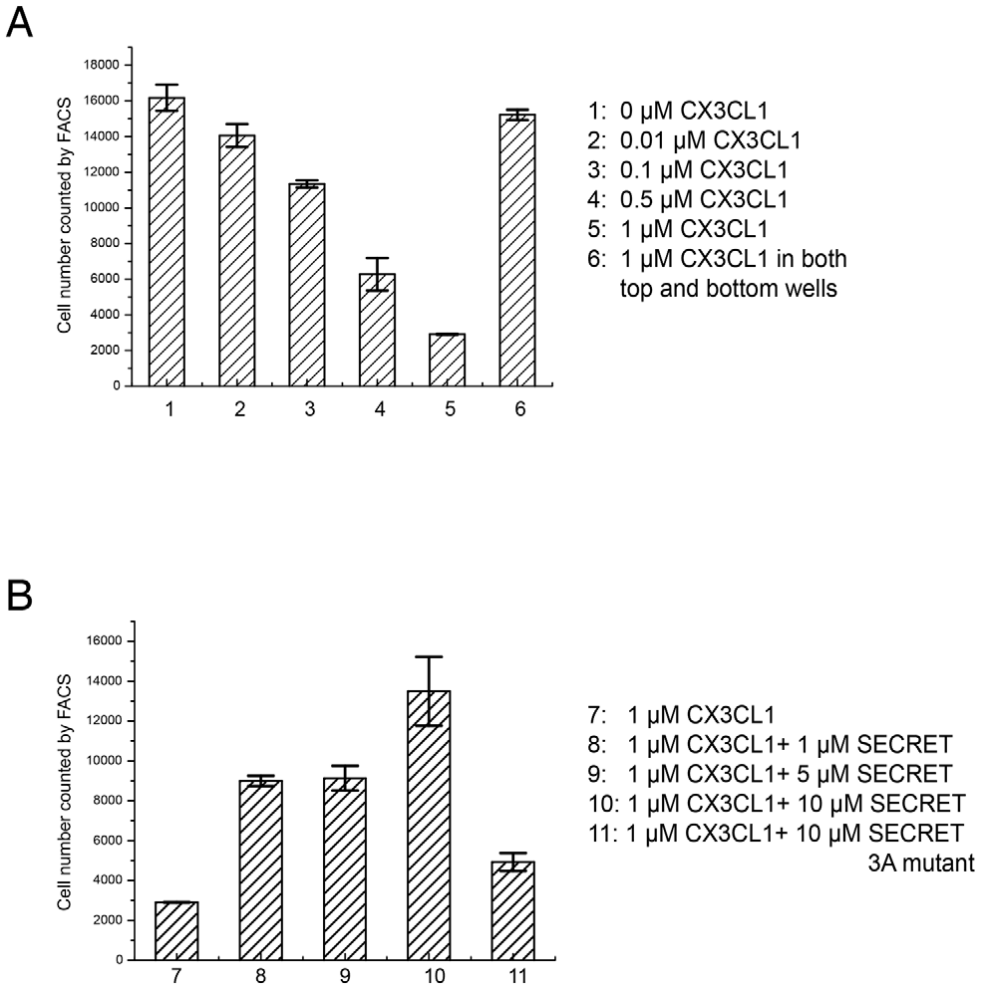
CX3CL1-induced migration of RAW 264.7 cells. (A) The number of cells remaining in the top well counted by FACS after a 4-h chemotaxis assay are indicated with CX3CL1 in concentrations of 0.01, 0.1, 0.5, and 1.0 µM in the bottom well, as well as the same concentration 1.0 µM in both top and bottom wells. (B) The number of cells remaining in the top well counted by FACS after a 4-h chemotaxis assay are indicated with CX3CL1, CX3CL1 pre-incubated with the SECRET domain in different molar ratio (1∶1, 1∶5, and 1∶10), and CX3CL1 pre-incubated with the SECRET domain triple mutant D167A/E169A/D316A in a 1∶10 molar ratio. The D167A/E169A/D316A mutant is referred as 3A mutant in the figure.

It has also been shown that the SECRET domain can inhibit the CCL25-mediated Molt4 cell migration, indicating its ability to interfere with binding of CCL25 with its cellular receptors [18], [30]. We also checked the ability of recombinant CX3CL1 in inducing migration of RAW264.7 cell as reported [43], as well as the ability of the SECRET domain to inhibit cell migration. CX3CL1 induced the migration of RAW264.7 cells in a dose-dependent manner, indicated by the decrease of cells remaining in the top well with the increase of CX3CL1 concentration in the bottom well (Figure 6A). Chemokinesis, defined as a random movement of cells in a zero gradient (equal amounts of starting chemoattractant in both top and bottom wells), was very low (Figure 6A). Pre-incubation of CX3CL1 with excessive SECRET domain significantly reduced the CX3CL1-mediated cell migration (Figure 6B). We also expressed and purified the SECRET domain with a triple mutation D167A/E169A/D316A by replacing its acidic residues involved in the critical salt-bridge interactions at the SECRET/CX3CL1 interface. Gel-filtration and circular dichroism (CD) spectroscopy profiles indicate that this mutant was properly folded and purified as the wild type protein (Figure S7 in Text S1). This SECRET domain mutant lost most of the inhibitory ability (Figure 6B). These results together suggest that the SECRET domain is able to interfere with the binding of CX3CL1 with its receptors on cell surface.

### The SECRET domain binds different chemokines similarly

**Figure 7:**
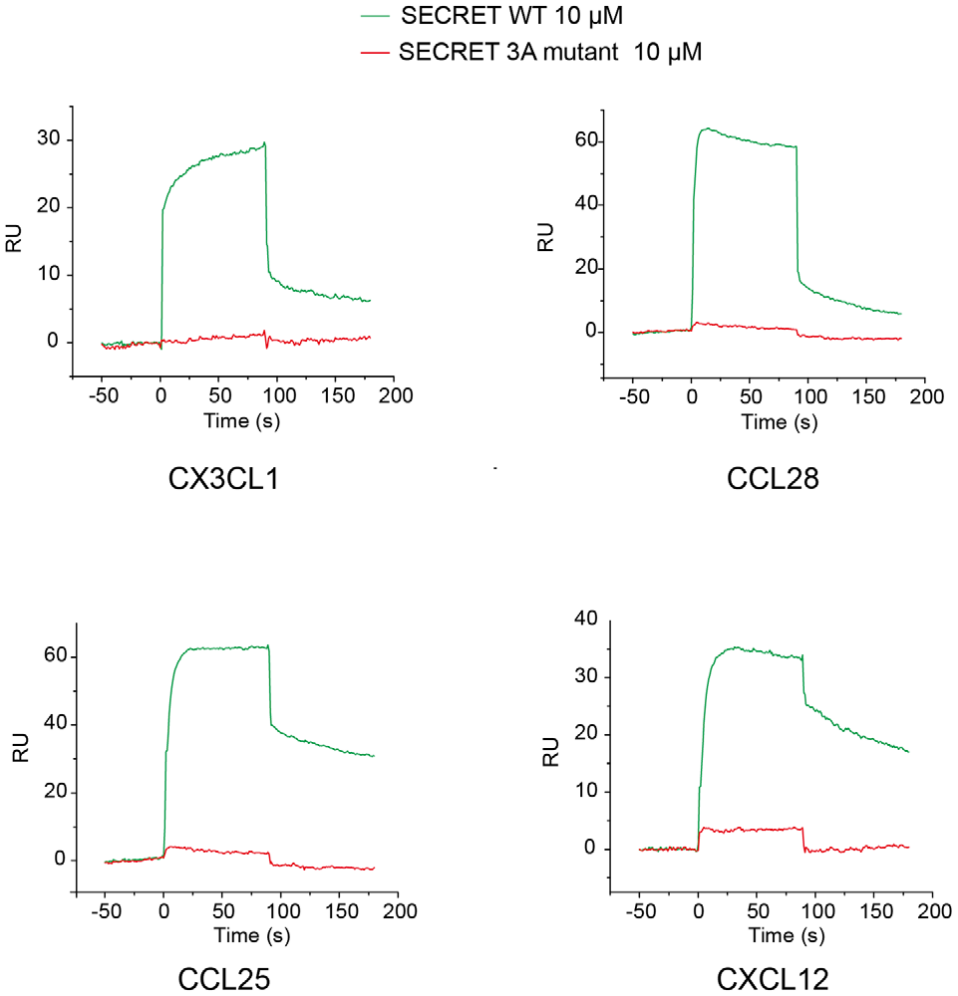
Sensograms of passing the SECRET domain wild type (green) and D167A/E169A/D316A mutant (red) through the CM5 chip surface immobilized with chemokines CX3CL1, CCL28, CCL25, and CXCL12, respectively. The D167A/E169A/D316A mutant is referred as 3A mutant in the figure.

The measured binding affinity (∼0.68 µM) between the SECRET domain and CX3CL1 in our experiment is lower than previous reported binding affinities between the SECRET domain and CCL28, CCL25, CXCL12, CXCL13, CXCL14, XCL1, and CCL20 that are in nM range [30]. This raises the question if the SECRET domain binds other chemokines in a manner similar to that observed in the SECRET/CX3CL1 complex structure. To help answer this question, we examined the binding ability of the SECRET domain D167A/E169A/D316A mutant. Besides CX3CL1, CCL28, CCL25 and CXCL12 were chosen because they were the previously reported top three in the binding with the CrmB and CrmD [30]. The SPR analysis showed that the triple mutations in the SECRET domain not only disrupted its binding with CX3CL1, but also with CCL28, CCL25, and CXCL12 to undetectable level (Figure 7), indicating that the SECRET domain binds different chemokines in a similar manner.

## Discussion

GAG binding plays important roles in the *in vivo* function of chemokines, including helping immobilize chemokines to form a concentration gradient along which cells can migrate directionally, protecting chemokines from proteolysis, and inducing chemokine oligomerization [44], [45]. It has been suggested that four distinct basic clusters on the surface of chemokines are major GAG-binding sites for different chemokines [8]. These four clusters all involve residues from the basic 40 loops. Residues on the chemokines important for GAG binding have also been characterized by mutagenesis studies for several chemokines including CCL2, CCL3, CCL4, CCL5, CXCL8, CXCL12, and XCL1 [6], [7]. Basic residues from the 40s loop participate in the binding of GAG by all studied chemokines except CXCL8 [6], [7]. The respective structures of CCL5 and CXCL12 with heparin-derived disaccharides also confirmed that the BBXB motif (where B and X stand for basic and neutral/hydrophobic amino acid) of the 40s loop participates in GAG binding [46], [47]. The inhibition of chemokine-GAG interaction by M3 is also attributed to its interaction with the basic 40s loop of bound chemokine by the NTD [23]. These previous results all suggest that the basic 40s loop of chemokines is generally involved in the GAG-binding. In the SECRET/CX3CL1 complex structure, the basic residues R44 and R47 from the 40s loop of CX3CL1 have direct interaction with the SECRET domain. We have also shown that heparin can interfere with the binding of CX3CL1 by the SECRET domain in a dose-dependent manner (Figure 5), similar to the interference of heparin in the chemokine binding of A41 and E163 [17], [18]. These data together indicate that the SECRET domain is able to block the chemokine-GAG interaction.

The inhibitory ability of the SECRET domain for CCL25 and CX3CL1 induced cell migration indicates that it is able to interfere with the chemokine-receptor interaction. It is generally accepted that the N-termini of chemokines is the key signaling domain, and other residues in the N-loop and core domain can also be critical for the binding with chemokine receptors. For example, the residues 12–17 in the N-loop of CXCL12 were shown to be important for receptor binding [47], [48]. The N-loop region (residues 13–20) of CC chemokines promotes tight binding to the chemokine receptors [49], [50]. The vCCI and M3 interfere with the chemokine-receptor interaction by completely blocking the accessibility of the N-loop region of bound chemokine, as revealed in the complex structure of vCCI with CCL4, and M3 with CCL2 and XCL1 [21], [23], [36]. The N-loop of CX3CL1 is not completely blocked by the SECRET domain. The S13 position at the N-terminal part of the N-loop critical for the binding of chemokines by vCCI and M3 is accessible in the SECRET/CX3CL1 complex, but residues T16, S17, K18, and I19 at the C-terminal part of the N-loop region have interaction with the SECRET domain. It suggests that although the SECRET domain does not directly block the most important receptor binding site on CX3CL1 (i.e. the N-termini and critical hydrophobic residues of the N-loop), its binding is still close to the receptor binding site and bring steric hindrance to prevent efficient interaction with the receptor, which would provide a structural basis for the ability of the SECRET domain to inhibit CX3CL1 and CCL25 induced cell migration.

The previous study reporting the discovery of the SECRET domain has shown that it is capable of binding CCL28, CCL25, CCL20, CXCL12, CXCL13, CXCL14 and XCL1 that are from the CC, CXC, and C classes. We have shown here that it is also able to bind CX3CL1, the only member in the CX3C class. The ability of previous reported M3 to bind a subset of chemokines from all four classes is attributed to its structural plasticity (i.e. the structural rearrangement of NTD and CTD) and the use of flexible loops as primary contact sites for chemokines from different classes [21], [23]. In comparison, the SECRET domain has a much smaller solvent exposed surface on the relatively rigid β-sheet I to contact chemokines from four different classes, demanding the focus on more common amino acid motifs on chemokines. In the SECRET/CX3CL1 complex structure, critical residues R44 and R47 for the complex formation are from the 40s loop, which can be regarded as hot-spot residues for the interaction. The presence of basic residues in the 40s loop is also found in other chemokines bound by the SECRET domain. The electrostatic complementary between the basic 40s loop of bound chemokine and acidic β-sheet I surface of the SECRET domain would enable the SECRET domain to bind different chemokines, allowing some extent of conformational variation in the 40s loop. There are two questions need to be answered in the future study: (1) Why is the SECRET domain not able to bind other chemokines also with the presence of basic residues in the 40s loop? (2) Why is the binding affinity of the SECRET domain with CX3CL1 lower than with previous reported chemokines? Sequence alignments of CX3CL1, CCL28, CCL25, CCL20, CXCL12, CXCL13, CXCL14, and XCL1 did not reveal obvious conserved motifs in the 40s loop (Figure S8A in Text S1) that are absent in chemokines unable to bind the SECRET domain. Previous NMR studies indicated that the flexibility of the N-loop is greater than the flexibility of other regions of chemokines (excluding the N- and C-termini) [51]. Only the C-terminal part of the N-loop of CX3CL1 is involved in the interaction with the SECRET domain. Due to the flexibility of the N-loop, it may more extensively participate in the interactions of CCL28, CCL25, CCL20, CXCL12, CXCL13, CXCL14 and XCL1 with the SECRET domain, and the chemokine selectivity of the SECRET domain may also reside in the flexible N-loop region. The definite and clear answers to these questions await future structural studies of the SECRET domain with chemokines from C, CC, and CXC classes.

Besides CrmB and CrmD, genome analysis also identified other genes encoding SECRET domain containing proteins (SCPs) [30]. The reported SCPs that bind to the same set of chemokines as CrmB and CrmD are CPXV V218 (SCP-1), ECTV E12 (SCP-2), and ECTV E184 (SCP-3) [30]. The primary sequence of the SECRET domain is much more conserved in CrmB and CrmD than in SCP-1, SCP-2, and SCP-3 (Figure S8B and S8C in Text S1). Among the fifteen residues in the SECRET domain of ECTV CrmD that have contacts with CX3CL1 in complex formation (Figure 4B and Figure S8B in Text S1), seven of them are strictly conserved in CrmB from VARV and CPXV and CrmD from ECTV and CPXV, including important charged residues D167 and E169 (Figure S8B in Text S1). Another important charged residue D316 is conserved in CrmD, but is replaced by arginine in CPXV CrmB and serine in VARV CrmB (Figure S8B in Text S1). Residues interacting with CX3CL1 in the SECRET domain are not highly conserved in SCP-1, SCP-2, and SCP-3 (Figure S8C in Text S1). This indicates that the binding of chemokines by these SCP proteins may be different from the binding by the SECRET domain.

## Methods

### Purification of the SECRET domain

The gene encoding the SECRET domain of CrmD (residues 162−320) was cloned into EcoRI and NcoI restriction sites of the pProEx HTb expression vector. The resulting plasmid was transformed into *E. coli* BL21 (DE3) competent cells. Three liters of LB media containing 100 µg/ml ampicillin were inoculated and grown to A_600_ of 0.8 and then induced with 0.6 mM IPTG. Induced cultures were grown for an additional 4 h at 37°C and harvested by centrifugation for 10 min at 5,000 rpm. Cells were resuspended in 25 mM Tris-HCl, 50 mM NaCl, pH 8.0, lysed with sonication and centrifuged for 50 min at 15,000 rpm. The SECRET domain was found exclusively in the inclusion bodies. The inclusion bodies were washed three times in wash buffer A (25 mM Tris-HCl, 50 mM NaCl, 5 mM EDTA, 5% Triton X-100, pH 8.0) and once in wash buffer B (25 mM Tris·HCl, 50 mM NaCl, 5 mM EDTA, pH 8.0). Washed inclusion bodies were solubilized in 8 M Urea, 50mM DTT and diluted into a refolding buffer (25 mM Tris-HCl, 50 mM NaCl, 0.2 mM oxidized glutathione, 2 mM reduced glutathione, pH 8.0) and stirred at 4°C overnight, and then dialyzed against 25 mM Tris-HCl, 50 mM NaCl, pH 8.0. The refolded SECRET domain was bound to HisTrap column, then washed with 25mM Tris-HCl, 50 mM NaCl, 20 mM Imidazole, pH 8.0 and eluted with 25 mM Tris-HCl, 50 mM NaCl, 500 mM imidazole, pH 8.0. Fractions containing the SECRET domain were examined by SDS-PAGE gel, pooled and further purified with size exclusion column. The SECRET mutant (D167A/E169A/D316A) was expressed and purified by the same method as wild type SECRET domain. To check the SECRET domain is a dimer or monomer in solution, molecular weight standards and the SECRET domain (0.5 ml, 1.5 mg/ml) were loaded onto Superdex 75 size exclusion column with a flow rate of 0.5 ml/min.

### Purification of CX3CL1 and the SECRET/CX3CL1 complex

The gene encoding the chemokine domain of human CX3CL1 (residues 1–76) was cloned into the EcoRI and NcoI restriction sites of the pProEX HTb expression vector. The resulting plasmid was transformed into *E. coli* BL21 (DE3) competent cells. Three liters LB media containing 100 µg/ml ampicillin were inoculated and grown to A_600_ of 0.8 and then induced with 1.0 mM IPTG. Induced cultures were grown for an additional 4 h at 37°C and harvested by centrifugation for 10 min at 5,000 rpm. Cells were resuspended in PBS buffer (pH 7.2), lysed by sonication and centrifuged for 50 min at 15,000 rpm. CX3CL1 was found exclusively in the inclusion bodies. The inclusion bodies were washed three times in wash buffer A (25 mM Tris·HCl, 50 mM NaCl, 5 mM EDTA, 5% Triton X-100, pH 8.0) and once in wash buffer B (25 mM Tris·HCl, 50 mM NaCl, 5 mM EDTA, pH 8.0). Washed inclusion bodies were solubilized in 8 M Urea, 50 mM DTT and diluted into a refolding buffer (PBS, 0.2 mM oxidized glutathione, 2 mM reduced glutathione, pH 7.2) and stirred at 4°C overnight. Precipitated material was removed by filtration. Refolded protein was bound to a HisTrap column and washed with PBS buffer, 20 mM Imidazole then eluted with PBS buffer, 500 mM Imidazole. Fractions containing CX3CL1 were examined by SDS-PAGE gel, pooled and further purified with size exclusion column. All CX3CL1 mutants were expressed and purified by the same method as wild type CX3CL1. Purified SECRET domain and wild type CX3CL1 were mixed, left on ice for 1 h, and subjected to size exclusion column purification to obtain the SECRET/CX3CL1 complex.

### Crystallization and data collection

The SECRET domain and the SECRET/CX3CL1 complex were concentrated by ultrafiltration to ∼15 mg/ml. Crystals of the SECRET domain were grown from a mother liquor of 0.4 M Magnesium formate dehydrate, 0.1 M Bis-Tris propane, pH 7.0 with hanging-drop vapor diffusion method at room temperature. Crystals of the SECRET/CX3CL1 complex were grown from 0.2 M Lithium sulfate monohydrate, 0.1 M Tris·HC, pH 8.5, 20% PEG4000 with hanging-drop vapor diffusion method at room temperature.

Crystals of the SECRET domain were cryoprotected in well solution plus 20% (v/v) glycerol and cooled to 100 K before data collection. For the SAD data collection, crystals were soaked in well solution with 0.2 M NaBr for 30 s before data collection. Crystals of the SECRET/CX3CL1 complex were cryoprotected in well solution plus 20% (v/v) glycerol and cooled to 100 K before data collection. All diffraction data were collected at Shanghai Synchrotron Research Facility (SSRF) beamline BL17U. All data were indexed and integrated and scaled with program HKL2000 [52].

### Structure determination and refinement

The structure of the SECRET domain was solved using the Br-SAD method. The positions of the Br were determined using the program SHELXD [53] and initial phases computed with the program SHELXE [54] as part of the HKL2MAP package [55]. Density modification was conducted using DM from the CCP4 suite [56]. The resulting electron density map was of excellent quality, allowing an automatic chain trance to be performed with the program Arp/wARP [57]. The following model adjustment and structural refinement were conducted using the program COOT [58] and PHENIX [59], respectively. For the final model, the *R_work_* is 16.4%, and the *R_free_* is 19.9%. The structure of the SECRET/CX3CL1 complex was solved using the molecular replacement method with the SECRET domain and the CX3CL1 structures as search models in the program PHASER [60]. Iterative refinement with the program PHENIX [59] and model building with the program COOT [58] were conducted, yielding a final *R_work_* of 19.6% and *R_free_* of 25.0%. All structural figures were made by using PYMOL (http://www.pymol.org).

### SPR experiments

The binding affinity between the SECRET domain and CX3CL1 was determined by surface plasmon resonance (SPR) using BIAcore 3000 at 25°C. The SECRET domain was immobilized to about 350 Response Unit (RU) on a research-grade CM5 sensor chip in 10 mM sodium acetate, pH 4.1 by standard amine coupling method. The flow cell 1 was left blank as a reference. To measure binding affinity of CX3CL1 wild type and mutants by the SECRET domain, CX3CL1 in 10 mM HEPES, pH 7.2, 150 mM NaCl, and 0.005% Tween-20 were injected over the flow cells at different concentrations at a flow rate of 30 µl min^−1^. The binary complexes were allowed to associate for 90 s and dissociate for 90 s. The surfaces were regenerated with 5 mM NaOH between each cycle if needed. Data were analyzed with BIAcore 3000 evaluation software BIAevaluation 4.1.

To investigate the interference of heparin in the binding of CX3CL1 by the SECRET domain, 1 µM wild type CX3CL1 was pre-incubated with increasing concentrations (0.125, 0.25, 0.5, 1.0, 2.0, 4.0 mg/ml) of heparin sodium salt (MW ∼15,000 Da, Sigma-Aldrich) at 4°C for 1 h. SPR analysis was performed as above.

To compare the binding ability of the SECRET domain wild type and mutant by chemokines, CX3CL1 purified by ourselves, CCL28, CCL25, and CXCL12 purchased from PeproTech were immobilized on the CM5 chip to ∼200 RU. SPR analysis was performed as above.

### Chemotaxis assay

RAW 264.7 cells were cultured in RPMI 1640 medium supplemented with 10% heat-inactivated FBS at 37°C in CO_2_ incubator. Serum-starved RAW 264.7 cells with a total number of 1×10^7^ were suspended in PBS buffer with 1 µM CellTracker Green CMFDA (Invitrogen) and incubated at 37°C for 5 minutes. The labeled cells were collected, washed three times with PBS buffer to remove the excessive CMFDA, and then suspended in RPMI 1640 medium for cell migration assays.

Cell chemotaxis assay was performed using 8 µm−pore Cell Culture Inserts (Millipore). The inserts were placed into 24-well plates containing RPMI1640 in the presence or the absence of CX3CL1 and SECRET domain. We seeded 8×10^4^ CMFDA-labeled cells in each transwell insert and incubated at 37°C for 4 hours. Cell migration was quantified by counting the number of cells that remaining in the upper transwell by FACS.

### Accession numbers

The coordinates of the SECRET domain and SECRET/CX3CL1 structures have been deposited into the Protein Data Bank with accession numbers 3ON9 and 3ONA, respectively.

## Supporting Information

Text S1 Supporting data including eight supplemental figures.
(PDF)
