# Supplementary material for: Structural Basis of Chemokine Sequestration by CrmD, a Poxvirus-Encoded Tumor Necrosis Factor Receptor
Source: PLoS Pathog. 2011 Jul 28;7(7):e1002162. doi: 10.1371/journal.ppat.1002162 (PMC3145792; doi:10.1371/journal.ppat.1002162)

Supporting Data

Figure S1 Electron density map, contoured at 1.0 σ of the SECRET domain (A) and the SECRET/CX3CL1 complex (B). The SECRET domain is colored in green, whereas CX3CL1 is colored in purple.

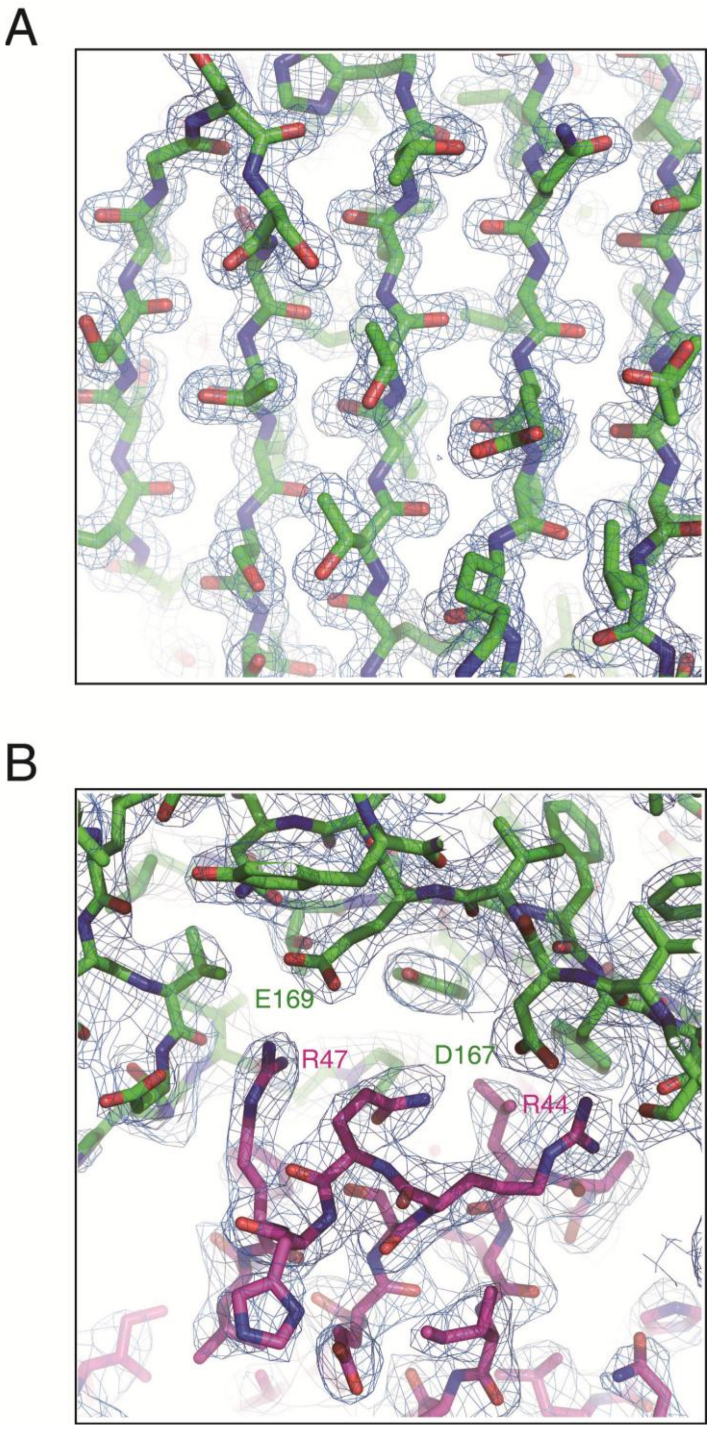

Figure S2 Size exclusion profile of the SECRET domain and molecular weight standards.

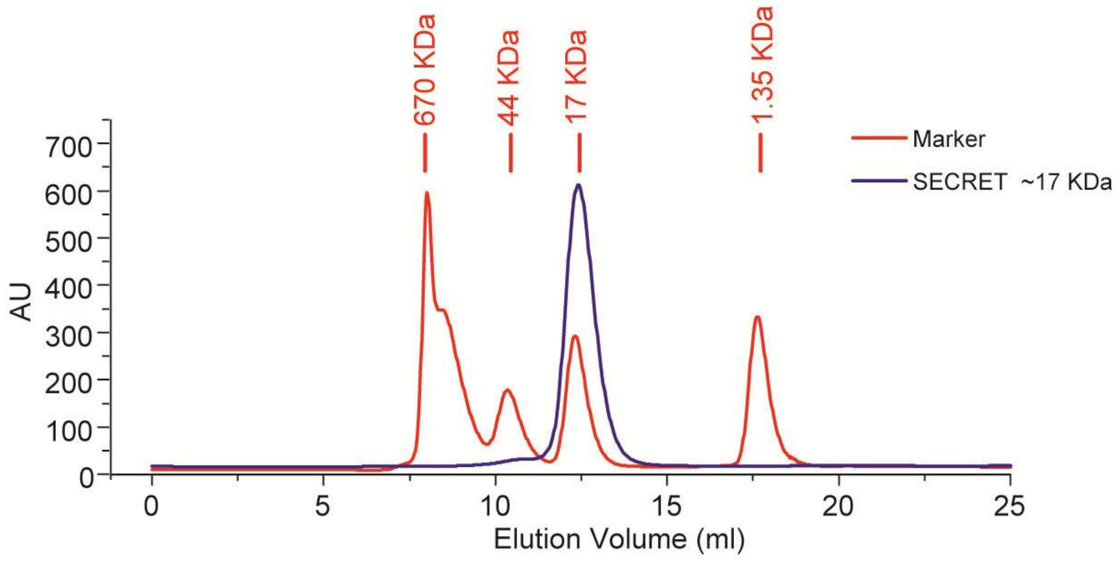

Figure S3 Topology diagram of the SECRET domain monomer. A dashed line separates the β-sheets I and II of the β-sandwich fold. The disulfide bonds are shown as orange bars.

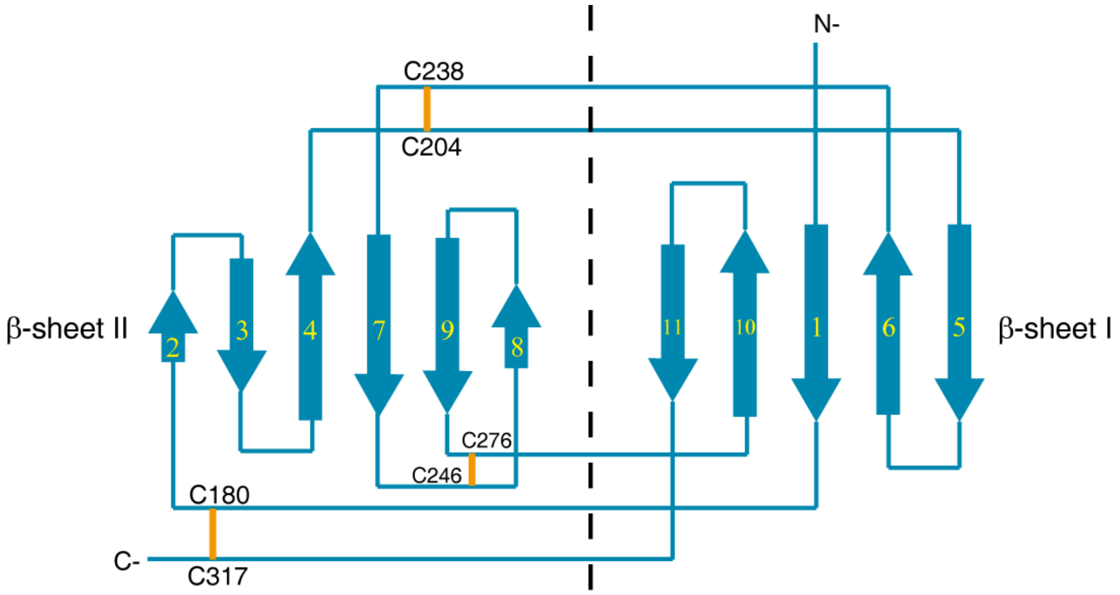

Figure S4 Sequence alignment of the SECRET domain, vCCI, and A41. The β-strands forming the β-sandwich fold of the SECRET domains are numbered from 1 to 11. Underlined are the 2-3 loop, 6-7 loop, and 7-9 loop. The acidic residues involved in the interactions with chemokines are colored as red.

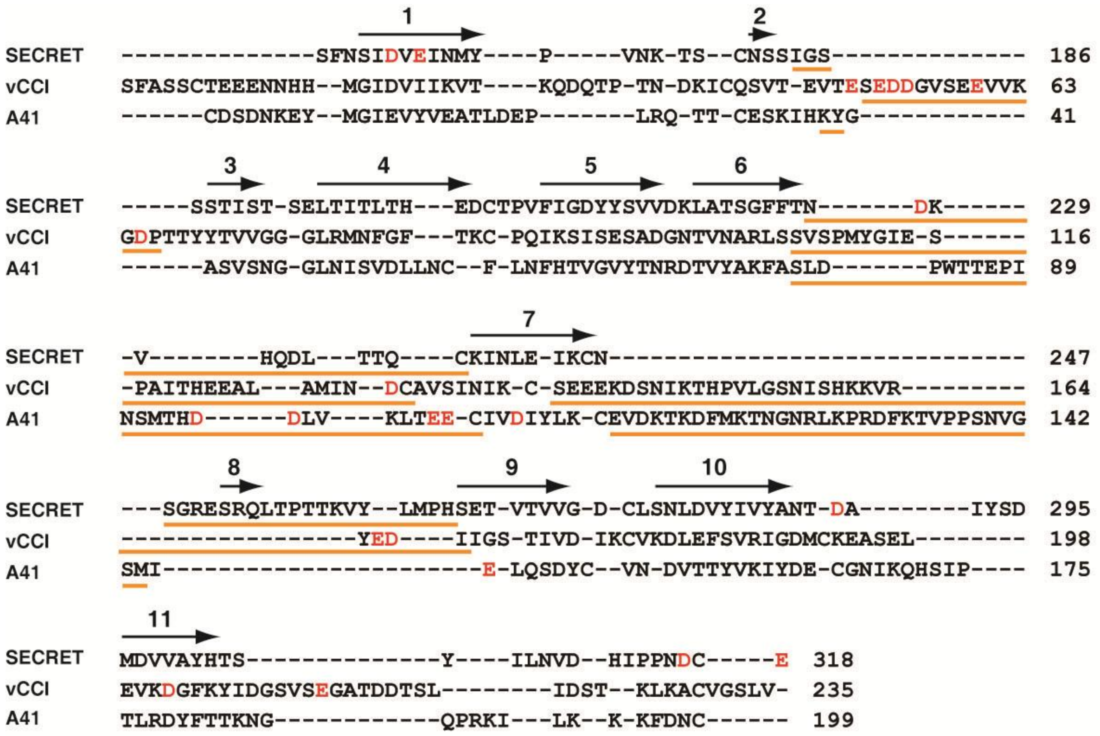

Figure S5 SPR sensograms for the binding of wild type CX3CL1 (A) and its mutants K18A (B), I19A (C), L23A (D), R44A (E), R47A (F), and F49A (G) by the SECRET domain. All measurements were conducted two times independently.

A

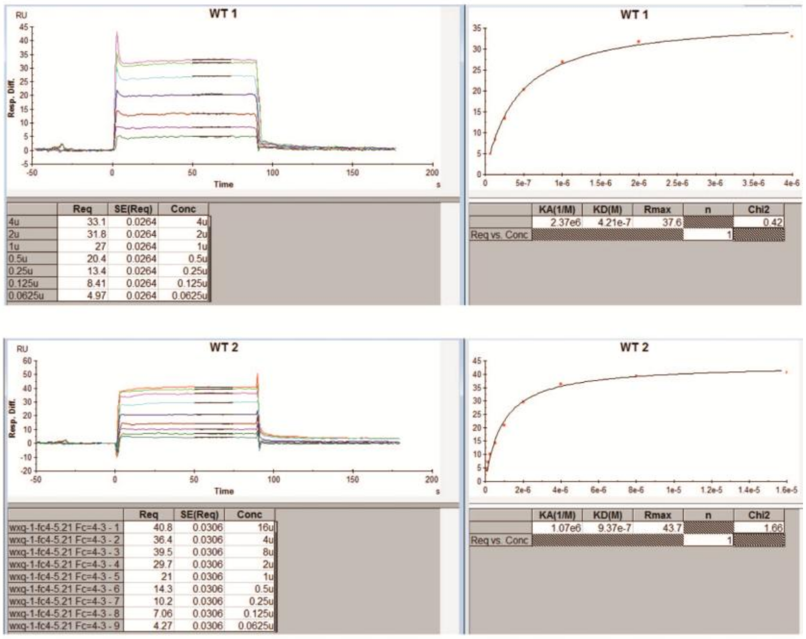

B

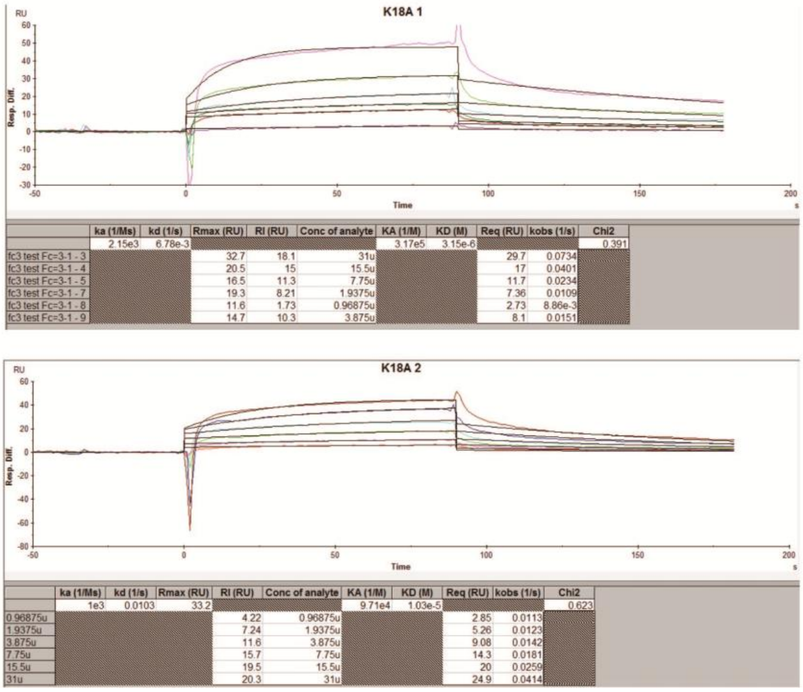

C

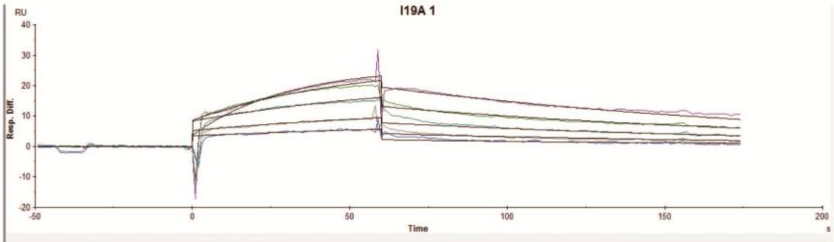

	ka (1/Ms)	kd (1/s)	Rmax (RU)	RI (RU)	Conc of analyte	KA (1/M)	KD (M)	Req (RU)	kobs (1/s)	Chi2
	5.38e3	6.89e-3	28.2			7.81e5	1.28e-6			0.145
5u				3.66	5u			22.5	0.0338	
2.5u				8.58	2.5u			18.7	0.0203	
1.25u				8.35	1.25u			13.9	0.0136	
0.625u				5.22	0.625u			9.26	0.0103	
0.3125u				3.47	0.3125u			5.54	8.57e-3	

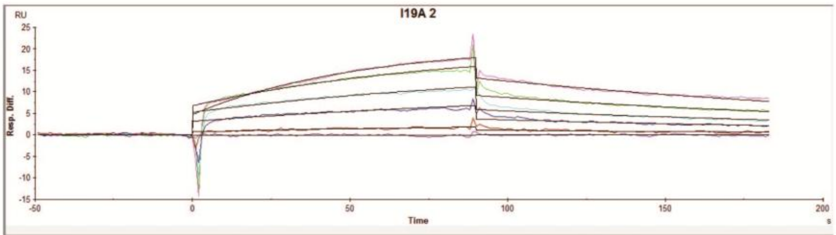

	ka (1/Ms)	kd (1/s)	Rmax (RU)	RI (RU)	Conc of analyte	KA (1/M)	KD (M)	Req (RU)	kobs (1/s)	Chi2
	5.33e3	5.7e-3				9.35e5	1.07e-6			0.0522
19a' Fc=4-3 - 2			23.1	4.77	2.5e-6			16.2	0.019	
19a' Fc=4-3 - 3			25.4	6.8	1.25e-6			13.7	0.0124	
19a' Fc=4-3 - 4			28.8	5.28	6.25e-7			10.6	9.03e-3	
19a' Fc=4-3 - 5			33.8	3.15	3.13e-7			7.63	7.37e-3	
19a' Fc=4-3 - 6			20.8	0.722	1.5e-7			2.56	6.5e-3	
19a' Fc=4-3 - 7			0.862	-0.0662	0			0	5.7e-3	

D

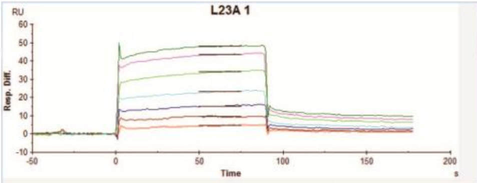

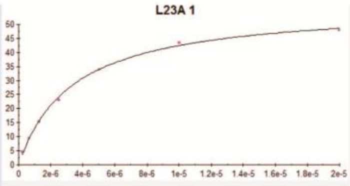

	Req	SE(Req)	Conc
0.2u	4.6	0.0531	0.2u
0.625u	43.5	0.0531	10u
1.25u	34	0.0531	5u
2.5u	23.1	0.0531	2.5u
5u	15.3	0.0531	1.25u
10u	9.45	0.0531	0.625u
20u	48	0.0531	20u

	KA(1/M)	KD(M)	Rmax	n	Chi2
	2.98e5	3.36e-6	56.7		0.986
Req vs. Conc				1	

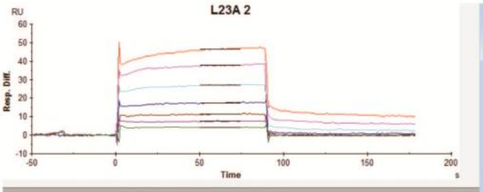

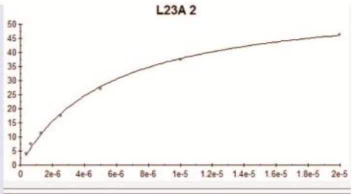

	Req	SE(Req)	Conc
wxq-1-fc4-5.21 Fc=4-3 - 11	46.5	0.0377	20u
wxq-1-fc4-5.21 Fc=4-3 - 12	37.6	0.0377	10u
wxq-1-fc4-5.21 Fc=4-3 - 14	27.1	0.0377	5u
wxq-1-fc4-5.21 Fc=4-3 - 15	17.5	0.0377	2.5u
wxq-1-fc4-5.21 Fc=4-3 - 16	11.4	0.0377	1.25u
wxq-1-fc4-5.21 Fc=4-3 - 17	7.43	0.0377	0.625u
wxq-1-fc4-5.21 Fc=4-3 - 18	4.15	0.0377	0.3125u

	KA(1/M)	KD(M)	Rmax	n	Chi2
	1.78e5	5.62e-6	59		0.996
Req vs. Conc				1	

E

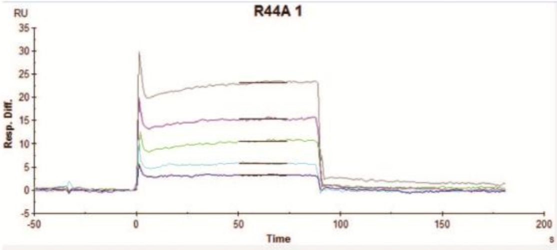

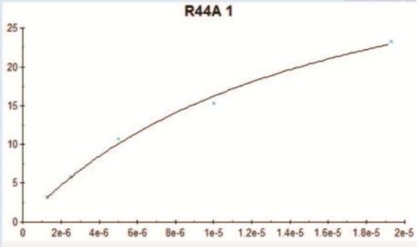

	Req	SE(Req)	Conc
wxq-1-fc4-5.20 Fc=4-3 - 26	10.7	0.0348	5u
wxq-1-fc4-5.20 Fc=4-3 - 27	5.79	0.0348	2.5u
wxq-1-fc4-5.20 Fc=4-3 - 28	3.24	0.0348	1.25u
wxq-1-fc4-5.20 Fc=4-3 - 30	15.3	0.0348	10u
wxq-1-fc4-5.20 Fc=4-3 - 33	23.3	0.0348	19.3u

	KA(1/M)	KD(M)	Rmax	n	Chi2
	6.37e4	1.57e-5	41.7		0.458
Req vs. Conc				1	

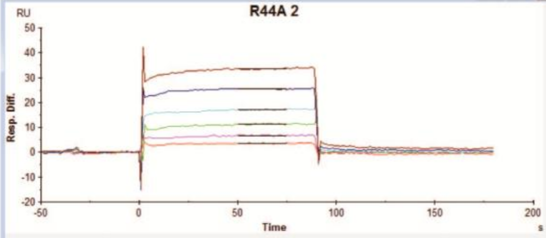

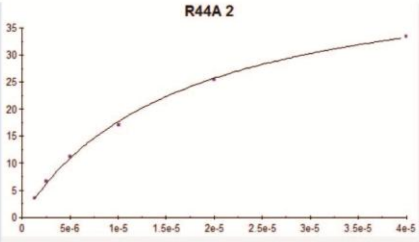

	Req	SE(Req)	Conc
wxq-1-fc4-5.21 Fc=4-3 - 22	3.54	0.0262	1.25u
wxq-1-fc4-5.21 Fc=4-3 - 23	6.7	0.0262	2.5u
wxq-1-fc4-5.21 Fc=4-3 - 24	11.3	0.0262	5u
wxq-1-fc4-5.21 Fc=4-3 - 25	17.1	0.0262	10u
wxq-1-fc4-5.21 Fc=4-3 - 26	25.5	0.0262	20u
wxq-1-fc4-5.21 Fc=4-3 - 27	33.5	0.0262	40u

	KA(1/M)	KD(M)	Rmax	n	Chi2
	6.04e4	1.66e-5	47		0.243
Req vs. Conc				1	

F

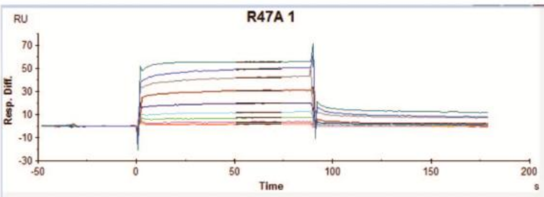

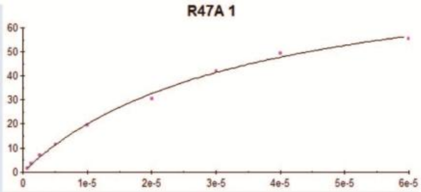

	Req	SE(Req)	Conc
wxq-1-fc4-5.21 Fc=4-3 - 42	1.69	0.0495	0.625u
wxq-1-fc4-5.21 Fc=4-3 - 43	3.47	0.0495	1.25u
wxq-1-fc4-5.21 Fc=4-3 - 44	7.1	0.0495	2.5u
wxq-1-fc4-5.21 Fc=4-3 - 45	11.8	0.0495	5u
wxq-1-fc4-5.21 Fc=4-3 - 46	19.6	0.0495	10u
wxq-1-fc4-5.21 Fc=4-3 - 47	30.7	0.0495	20u
wxq-1-fc4-5.21 Fc=4-3 - 57	55.6	0.0495	60u
wxq-1-fc4-5.21 Fc=4-3 - 63	42.2	0.0495	30u
wxq-1-fc4-5.21 Fc=4-3 - 64	49.6	0.0495	40u

	KA(1/M)	KD(M)	Rmax	n	Chi2
	2.9e4	3.45e-5	89.1		1.48
Req vs. Conc				1	

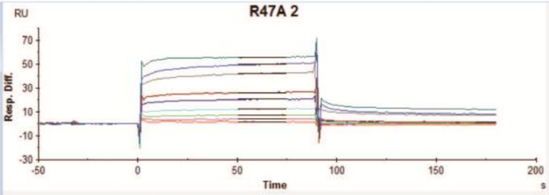

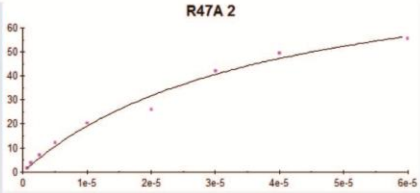

	Req	SE(Req)	Conc
wxq-1-fc4-5.21 Fc=4-3 - 49	1.58	0.0485	0.625u
wxq-1-fc4-5.21 Fc=4-3 - 50	3.88	0.0485	1.25u
wxq-1-fc4-5.21 Fc=4-3 - 51	7.29	0.0485	2.5u
wxq-1-fc4-5.21 Fc=4-3 - 52	12.3	0.0485	5u
wxq-1-fc4-5.21 Fc=4-3 - 53	20.5	0.0485	10u
wxq-1-fc4-5.21 Fc=4-3 - 54	26.1	0.0485	20u
wxq-1-fc4-5.21 Fc=4-3 - 57	55.6	0.0485	60u
wxq-1-fc4-5.21 Fc=4-3 - 63	42.2	0.0485	30u
wxq-1-fc4-5.21 Fc=4-3 - 64	49.6	0.0485	40u

	KA(1/M)	KD(M)	Rmax	n	Chi2
	2.6e4	3.84e-5	92.7		6.92
Req vs. Conc				1	

G

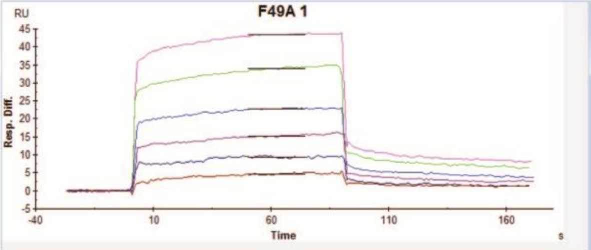

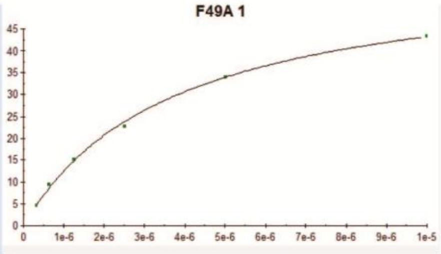

	Req	SE(Req)	Conc
49a Fc=4-3 - 5 10u	43.4	0.0474	10u
49a Fc=4-3 - 6 5u	34	0.0474	5u
49a Fc=4-3 - 7 2.5u	22.8	0.0474	2.5u
49a Fc=4-3 - 8 1.25u	15.2	0.0474	1.25u
49a Fc=4-3 - 9 0.625u	9.38	0.0474	0.625u
49a Fc=4-3 - 4 0.3125u	4.68	0.0474	0.3125u

	KA(1/M)	KD(M)	Rmax	n	Chi2
	2.67e5	3.75e-6	59.4		0.475
Req vs. Conc				1	

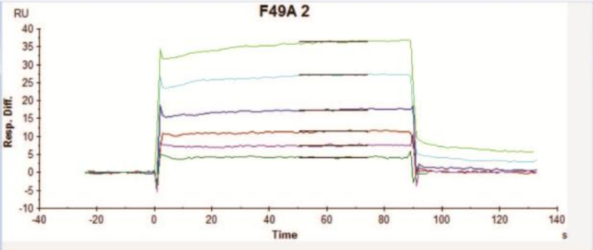

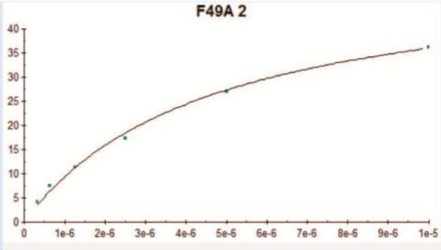

	Req	SE(Req)	Conc
49a' Fc=4-3 - 13 10u	36.3	0.034	10u
49a' Fc=4-3 - 14 5u	27.1	0.034	5u
49a' Fc=4-3 - 15 2.5u	17.4	0.034	2.5u
49a' Fc=4-3 - 16 1.25u	11.4	0.034	1.25u
49a' Fc=4-3 - 17 0.625u	7.5	0.034	0.625u
49a' Fc=4-3 - 18 0.3125u	4.14	0.034	0.3125u

	KA(1/M)	KD(M)	Rmax	n	Chi2
	2.15e5	4.64e-6	52.7		0.878
Req vs. Conc				1	

Figure S6 Structural comparison of the SECRET/CX3CL1 complex with vCCI/CCL4, Evasin-1/CCL3, and M3/CCL2 complexes. (A) Left panel shows the contact patch 1 at the vCCI/CCL4 binding interface. Middle panel shows the contact patch 2 at the vCCL/CCL4 binding interface. Right panel shows the superimposed SECRET/CX3CL1 and vCCI/CCL4 complexes based on bound chemokines. (B) Left panel shows the Evasin-1/CCL3 complex. Right panel shows the superimposed SECRET/CX3CL1 and Evasin-1/CCL3 complexes based on bound chemokines. (C) Right panel shows the M3/CCL2 complex. Middle panel shows the superimposed SECRET/CX3CL1 and M3/CCL2 complexes based on bound chemokines. Right panel shows the superimposed SECRET/CX3CL1, Evasin-1/CCL3, and M3/CCL2 complexes based on bound chemokines.

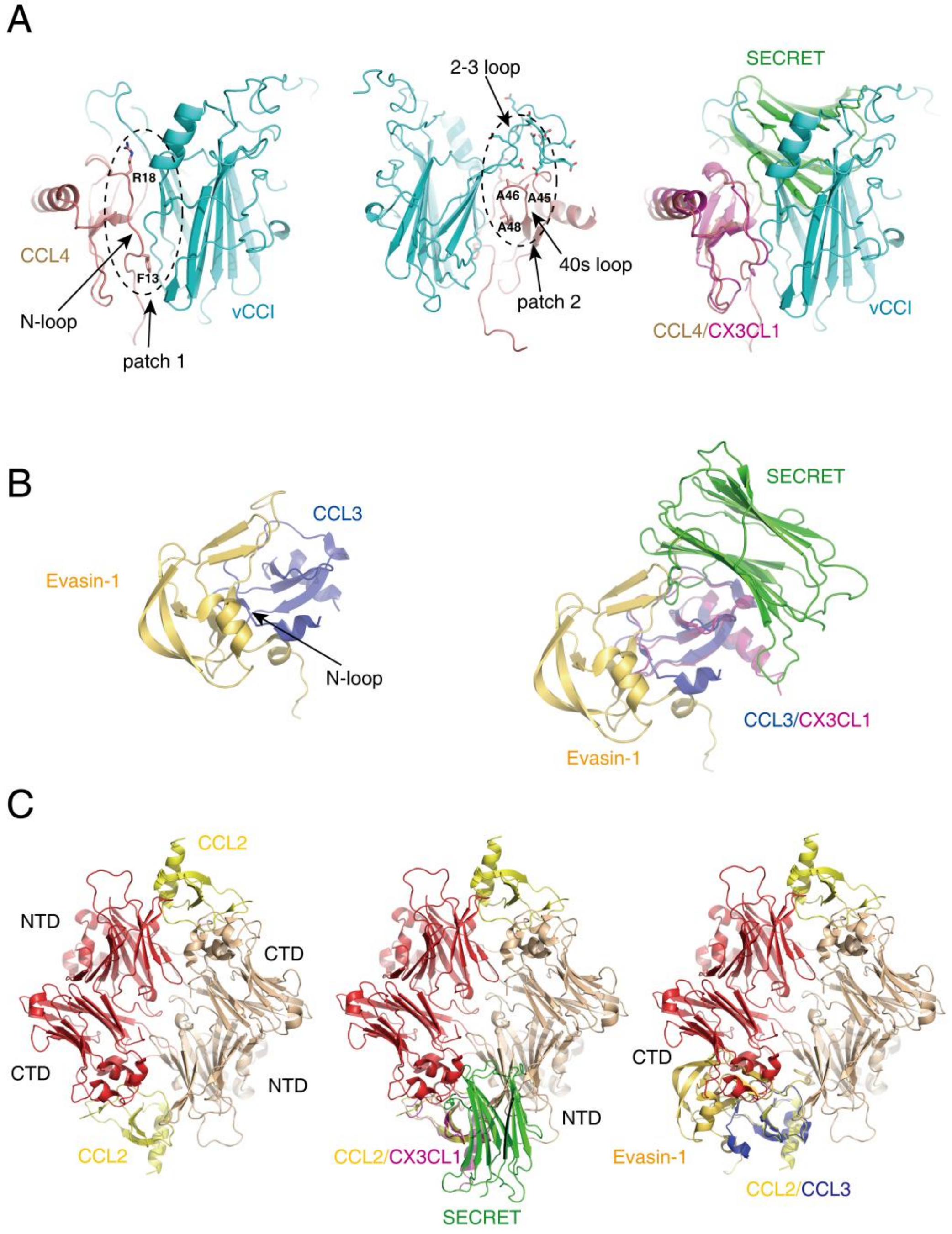

Figure S7 Gel filtration profile (A) and CD curve (B) of the SECRET domain wild type and its D167A/E169A/D316A mutant. The SECRET domain D167A/E169A/D316A mutant is referred as 3A in the figure.

A

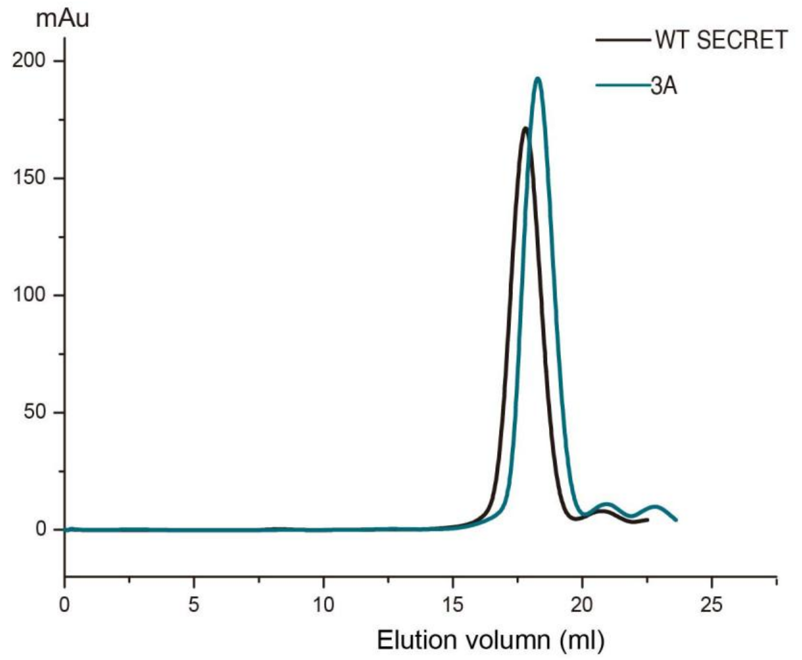

B

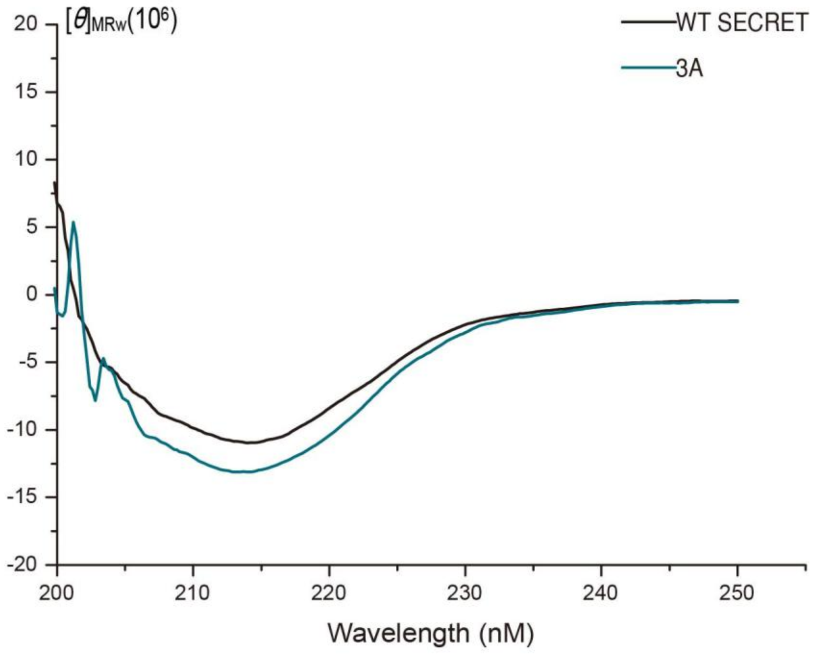

Figure S8 Sequence alignments. (A) Alignment of chemokines CX3CL1, CCL28, CCL25, CCL20, CXCL12, CXCL13, CXCL14, and XCL1. (B) Alignment of the SECRET domain from CrmB and CrmD. Blue triangles label residues involved in the interaction with CX3CL1. Red circles label interacting residues that are strictly conserved in CrmB and CrmD. (C) Alignment of the SECRET domain from ECTV CrmD with SCP proteins.

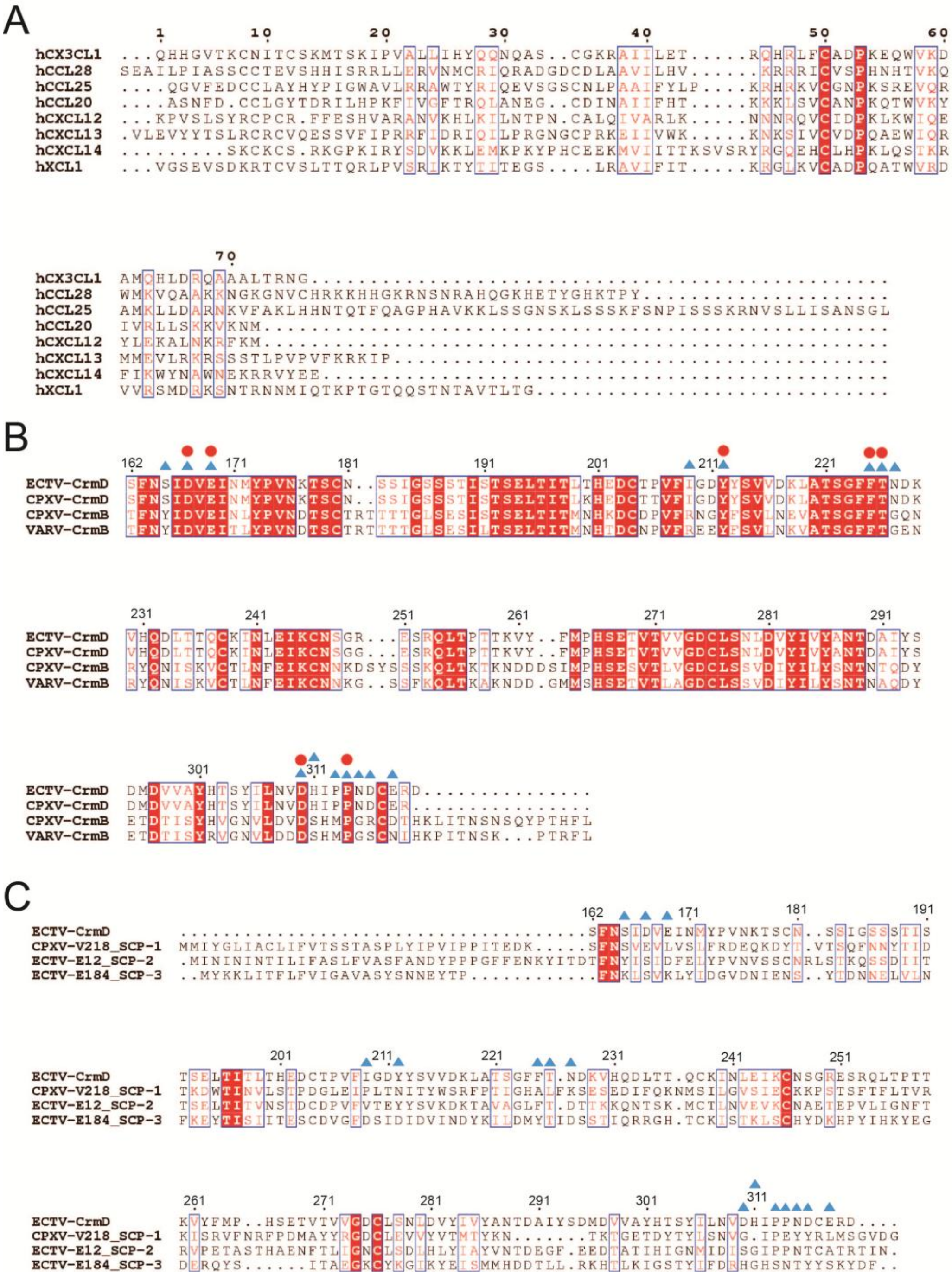

Supplement: Text S1 — Supporting data including eight supplemental figures. (PDF) [file ppat.1002162.s001.pdf]
